# The prognostic significance of tumor-infiltrating lymphocytes assessment with hematoxylin and eosin sections in resected primary lung adenocarcinoma

**DOI:** 10.1371/journal.pone.0224430

**Published:** 2019-11-19

**Authors:** Ahrong Kim, So Jeong Lee, Jihyun Ahn, Won Young Park, Dong Hoon Shin, Chang Hun Lee, Hoon Kwon, Yeon Joo Jeong, Hyo Yeong Ahn, Hoseok I, Yeong Dae Kim, Jeong Su Cho

**Affiliations:** 1 Department of Pathology, Pusan National University Hospital, Biomedical Research Institution, Gudeok-ro, Seo-Gu, Busan, Republic of Korea; 2 Department of Pathology, Kosin University Gospel Hospital, Gamcheon-ro, Seo-gu, Busan, Republic of Korea; 3 Department of Pathology, Yangsan Pusan National University Hospital, Beomeori, Mulgeum-eop, Kyeong-Nam, Republic of Korea; 4 Department of Radiology, Pusan National University Hospital, Biomedical Research Institution, Gudeok-ro, Seo-Gu, Busan, Republic of Korea; 5 Department of Thoracic and Cardiovascular Surgery, Pusan National University Hospital, Biomedical Research Institution, Gudeok-ro, Seo-Gu, Busan, Republic of Korea; Baylor College of Medicine, UNITED STATES

## Abstract

The prognostic significance of tumor-infiltrating lymphocytes has been determined in cancers of the lung, colon and breast, though there is no standardized method for using this prognostic indicator for lung cancer. We applied a modified version of the method proposed by the International Immuno-Oncology Biomarkers Working Group to primary lung adenocarcinoma, which uses histologic findings of hematoxylin and eosin sections. The study included a total cohort of 146 lung adenocarcinoma patients who underwent lobectomy with lymph node dissection at two hospitals between 2008 and 2012. The full-face sections of hematoxylin and eosin-stained slides were reviewed, and we evaluated the level of tumor-infiltrating lymphocytes as a percentage of the area occupied out of the total intra-tumoral stromal area. Histopathologic factors include histologic grade, necrosis, extracellular mucin, lymphovascular invasion, lymph node metastasis, level of tumor infiltrating lymphocytes, tertiary lymphoid structures around the tumor, and the presence of a germinal center in tertiary lymphoid structures. The high level of tumor-infiltrating lymphocytes was found to be significantly correlated with the histologic grade (p = 0.023), necrosis (p = 0.042), abundance of tertiary lymphoid structures(p<0.001) and presence of a germinal center in tertiary lymphoid structures (p = 0.004). A high level of tumor-infiltrating lymphocytes was associated with better progression-free survival (p = 0.011) as well as overall survival (p = 0.049). On multivariable analysis, high tumor-infiltrating lymphocyte levels were a good independent prognostic factor for progression-free survival (Hazard ratio: 0.389, 95% confidence interval: 0.161–0.941, p = 0.036). Histologic evaluation of tumor-infiltrating lymphocytes level in lung adenocarcinoma with H&E sections therefore has prognostic value in routine surgical pathology.

## Introduction

Remarkable advances in immunotherapy have resulted in recent increased interest in cancer immunology. The immune system is now believed to have an important role in cancer development through “cancer immunoediting”, encompassing three processes: elimination, equilibrium, and escape [1, 2], and various studies support the role of immunosurveillance in lung cancer. Immune-mediated paraneoplastic syndromes in malignant tumors occur most frequently in lung cancer [3]. Organ transplant recipients, who are immunosuppressed, have a higher risk of developing non-small cell lung cancers [4]. Furthermore, Ichiki et al. reported that in the case of lung cancer, the immune system spontaneously recognized the tumor-associated antigens [5]. Also, the immune microenvironment in NSCLC is known to have a strong prognostic impact [6].

The prognostic significance of tumor-infiltrating lymphocytes (TILs) has been determined in various tumors, including cutaneous melanoma, breast cancer, and colon cancer. In addition, there are standardized methods for evaluating TILs in colon cancer and breast cancer [7, 8]. In colon cancer, the consensus Immunoscore is a reliable estimate of the risk of recurrence, which is a scoring system that represents the density of CD3+ and CD8+ T cells within the tumor along with the invasive margin [8]. The mean of the four percentiles obtained for each marker at either the tumor center or at invasive margins was calculated and translated into the Immunoscore scoring system. The final immunoscore was subdivided into 3 groups based on mean percentile: Immunoscore Low (0–25%), Intermediate (25–70%) and High (70–100%). The International TILs Working Group made a recommendation for evaluation of TILs in breast cancer. In brief, TILs should be estimated as a percentage for the stromal compartment within the tumor border [7]. All mononuclear cells encompassing lymphocytes and plasma cells should be scored, and granulocytes and other polymorphonuclear leukocytes should be excluded. Immunohistochemistry to assess the subtyping lymphocytes is not recommended outside of a research setting. The cutoff of stromal lymphocytes for lymphocyte predominant breast cancer is about 50–60% of the tumor stromal area [7].

Though many studies tried to delineate the prognostic significance of TILs in lung cancer, there had been no standardized consensus for TILs evaluation. In this background, the International Immuno-Oncology Biomarkers Working Group proposed the standardized method for TILs assessment in NSCLC [9].

In this study, we modified the TILs evaluation method proposed by the International Immuno-Oncology Biomarkers Working Group, then applied it to primary lung adenocarcinomas to evaluate its prognostic impact.

## Materials and methods

### Patients and tissue specimens

This study was performed retrospectively and included patients with primary lung adenocarcinoma who underwent resection with lymph node dissection between 2008 and 2012 at Pusan National University Hospital and Yangsan Pusan National University Hospital. Patients who had pre-operative chemo- or radiotherapy history were excluded, so all subjects were all pre-operatively chemo- and radiotherapy naïve. The final cohort was composed of 146 patients. Surgically resected specimens were cut along the bronchus and fixed in 10% buffered formalin overnight. For tumors which were grossly 3.0 cm or less in size whole tumor tissue was embedded for H&E sections, and in cases of those larger than 3.0 cm, 1 section per extra 1 cm was made additionally. All available hematoxylin and eosin (H&E) stained slides, which were made at the time of diagnosis, were used. Clinicopathological data were retrieved from the electric medical records and pathologic reports. Exemption from informed consent after de-identification of the patients’ information was approved by the Institutional Review Board of Pusan National University Hospital (H-1802-018-064) and Pusan National University Yangsan Hospital (05-2019-127).

### Histologic evaluation

All available full-face H&E sections were used. Two pathologists (A Kim and DH Shin) reviewed the H&E sections using a multi-head microscope to result in consensus. Histopathological factors included histologic grade, necrosis, extracellular mucin production, lymphovascular invasion, lymph node metastasis, tertiary lymphoid structures (TLSs) around the tumor, a germinal center in TLSs, and TILs level. The histologic grade was based on the predominant subtype among five histological patterns, including lepidic, acinar (including cribriform), papillary, solid and micropapillary patterns [10, 11].

**Fig 1:**
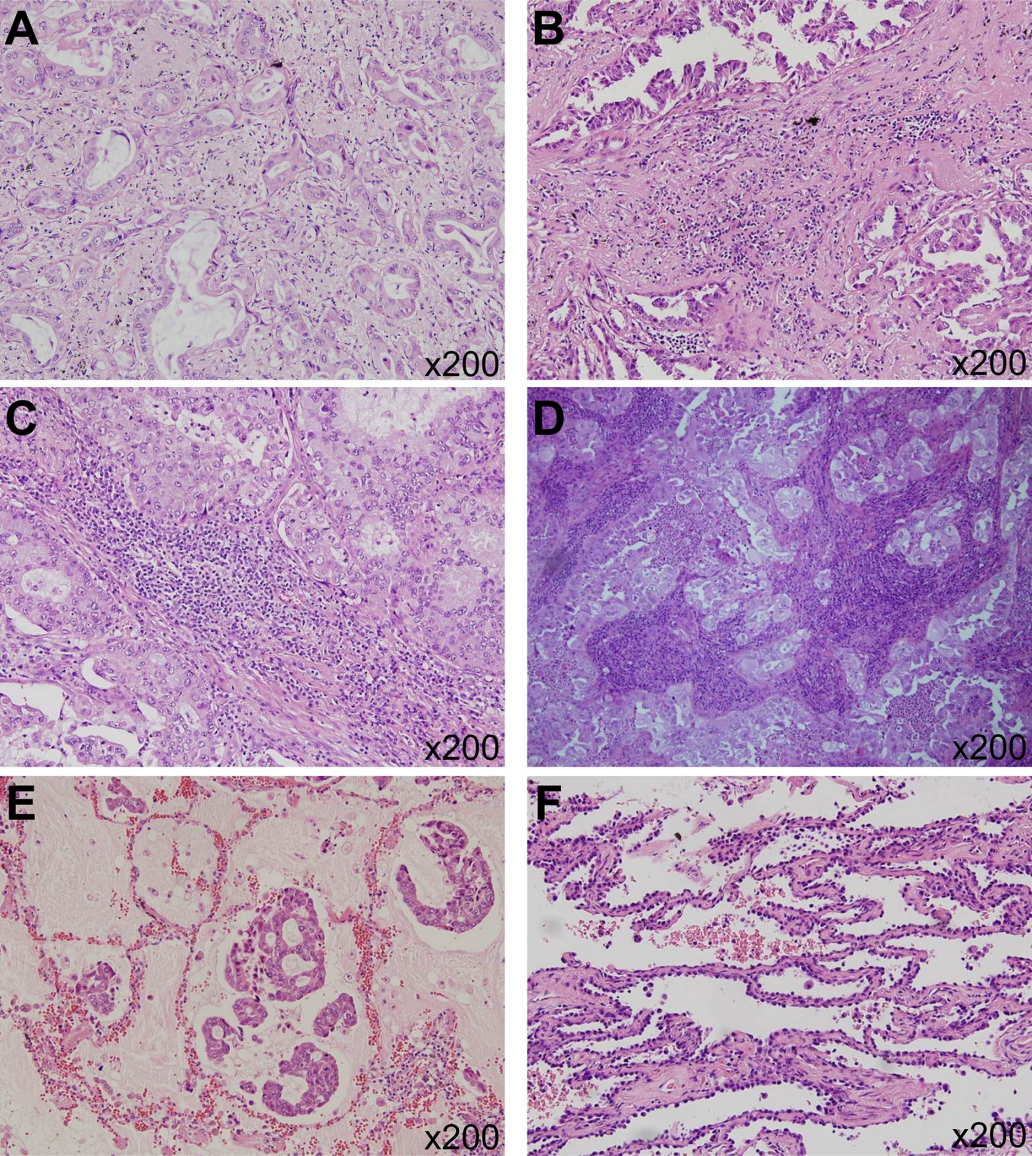
Assessment of tumor-infiltrating lymphocytes (TILs) level. (A) 5% of TILs (x200) (B) 20% of TILs (x200) (C) 60% of TILs (x200) (D) 80% of TILs (x200) (E) Area with aerosol spread was excluded. (x200) (F) Area with lepidic growth pattern was not included in assessment of TILs. (x200).

The method of TILs evaluation was primarily based on the guidelines from International Immuno-Oncology Biomarkers Working Group [9]. We assessed TILs level within the borders of the invasive tumor (in other words, within the stromal-tumor borderline), and areas within the tumor and particular area within the tumor was excluded in TILs evaluation. Area with necrosis, crush artifacts, fibrosis, and prior biopsy sites were excluded for TILs evaluation. Also, the peri-bronchial area was avoided in evaluating TILs, because bronchus-associated lymphoid tissue (BALT) originally resides in close proximity to the basal side of the bronchial epithelium [12]. Also, areas with aerogenic spread, which has no desmoplasia, and areas showing pure lepidic or intra-alveolar growth patterns without desmoplastic reaction were not included. However, stroma in fibrovascular cores of papillary structures were included. The invasive margin and the center of tumor were not separately considered and TILs within the tumor border (stromal-tumor borderline) were evaluated. A full assessment of average TILs level within the tumor border using all available full-face H&E sections was performed, so that the hotspots were included but not focused. TILs were reported for the stromal compartment within the tumor border. However, intra-tumoral TILs, which are TILs in tumor nests showing cell-to-cell contact with no intervening stroma and directly contacting tumor cells, were not considered. For the percentage of stromal TILs the area of stromal tissue, not the number of stromal cells, was used as a denominator. The level of TILs was calculated according to the percentage of stroma in the invasive area and was evaluated as a percentage in 10% increments. In cases of TILs level of less than 10%, a 1% or 5% criteria was applied (Fig 1). All mononuclear cells including lymphocytes and plasma cells were scored. Also, granulocytes and other polymorphonuclear leukocytes, especially intra-alveolar inflammatory cells including alveolar macrophages, were excluded from the TILs evaluation.

**Fig 2:**
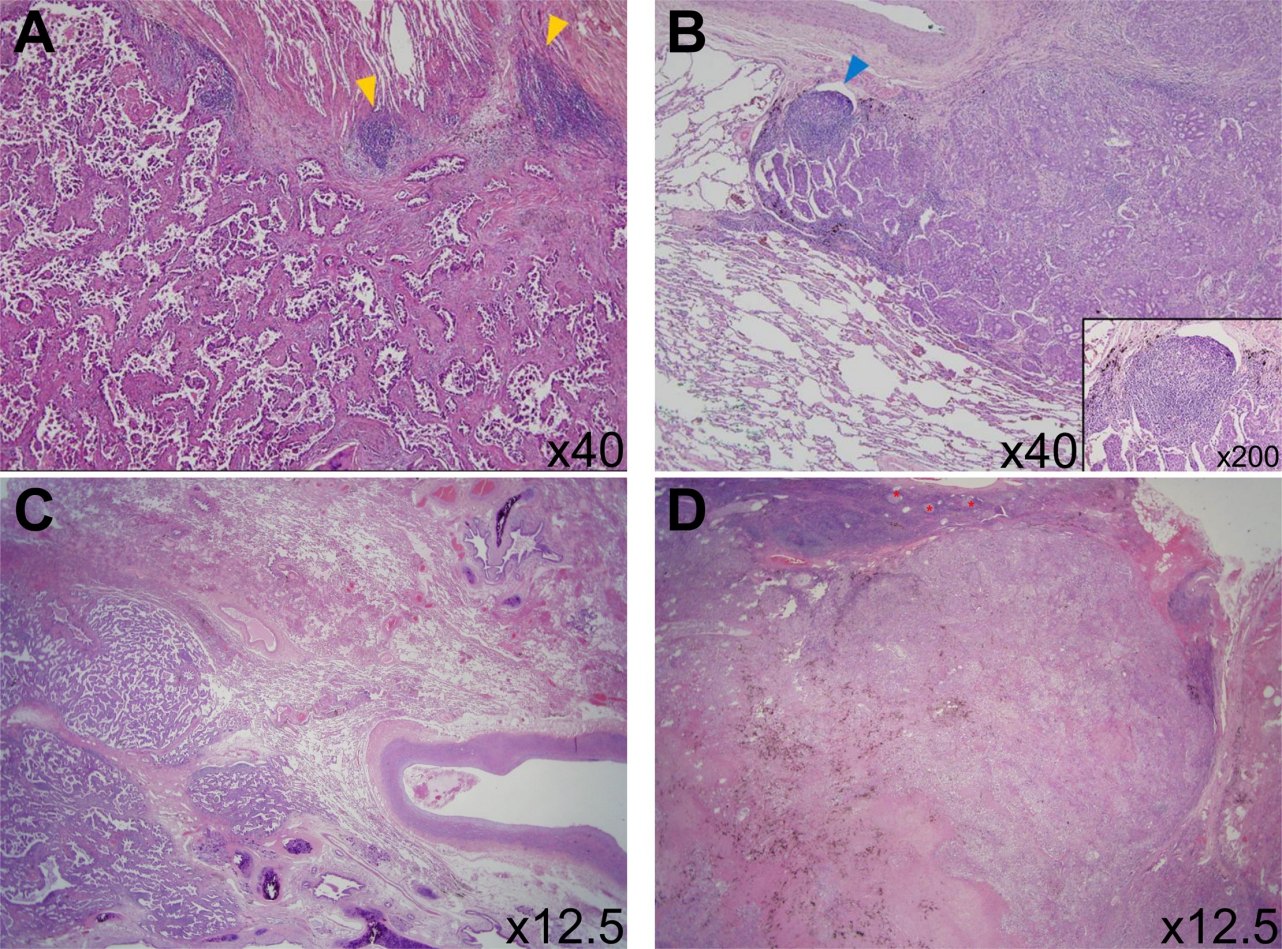
Assessment of tertiary lymphoid structures (TLSs). (A) Yellow colored arrow head indicates TLSs without germinal center around invasive tumor. (x40) (B) Blue colored arrow head indicates TLS with germinal center around invasive tumor (x40) and inset shows the magnified image of TLS (x200) (C) Absence of TLSs around invasive tumor (x12.5) (D) Abundant TLSs with germinal center (red asterick) around invasive tumor (x12.5).

TLS is a lymph node-like structure that is ectopic aggregation of lymphoid cells with specialized high endothelial venules (Fig 2). We evaluated the amount of TLSs as a percentage of the total circumference of the tumor front (stromal-tumor borderline) [13].

The cutoff for lymphocyte-predominant breast cancer is about 50–60%, but there is no standardized cutoff for lymphocyte-predominant lung cancer [7]. For statistical analysis, patients were subdivided into 2 categories (≥ 50% and <50%) according to arbitrary TILs level of 50%. The level of TLSs was categorized into 2 subgroups (low and high) based on the mean value.

### Statistical analysis

SPSS statistical software (version19: IBM, Armonk, NY, USA) was utilized for all statistical analyses. The Chi-square test, two-tailed test, Fisher’s exact test, Spearman rank correlation test, Cox proportional hazards regression model, log-rank test and Kaplan-Meier survival analyses were used as appropriate. Overall survival (OS) was defined as the time from the day of diagnosis until the death of the patient as determined by Kaplan-Meier survival curves. Progression-free survival (PFS) was defined as the time from the day of diagnosis until any event including additional metastasis, recurrence or an increase in the size of pre-existing lesion, and death by Kaplan-Meier survival curves. For all tests, a p-value of less than 0.05 was considered to be statistically significant.

## Results

### Clinicopathologic characteristics of primary lung adenocarcinomas

A cohort of 146 patients with primary lung adenocarcinoma was included, composed of 77 females and 69 male patients. Patient age ranged from 35 to 85 years (mean ± standard deviation: 63.73 ± 10.176 years) and the size of the lung adenocarcinoma ranged from 0.4 to 7.2 cm (mean ± standard deviation: 2.82 ± 1.319cm). One hundred twenty patients (82.2%) had early stage (stage I and II) lung cancer, and the other 26 patients (17.5%) had lung cancer of advanced stage (stage III and IV). Thirty-three patients (22.6%) were categorized as high TILs level, whereas the other 113 patients (77.4) had low TILs level. The amount of TLSs ranged from 0% to 60% (mean ± standard deviation: 4.68 ± 9.197%). Forty patients (27.4%) had high TLSs around tumor and 106 patients (72.6%) had low TLSs around the tumor. Also, 17 patients (11.6%) had a germinal center in TLSs and 129 patients (88.4%) had no germinal center in TLSs (Table 1).

**Table 1:** Basic data of primary lung adenocarcinoma.

Characteristic	Number (%)*
Age, mean ± SD	63.730 ± 10.176 years
Size, mean ± SD	2.820 ± 1.319 cm
Smoking history	
Never-smoker	85 (58.2)
Smoker	61 (41.8)
Histologic grade	
Well differentiated	111 (76.0)
Moderately differentiated	27 (18.5)
Poorly differentiated	8 (5.5)
Necrosis in tumor	
Absent	109 (74.7)
Present	37 (25.3)
Extracellular production	
Absent	123 (84.2)
Present	23 (15.8)
Lymphovascular invasion	
Absent	121 (82.9)
Present	25 (17.1)
Lymph node metastasis	
Absent	111 (76.0)
Present	35 (24.0)
Stage (AJCC 7^th^ edition)	
I	95 (65.1)
II	25 (17.1)
III	13 (8.9)
IV	13 (18.9)
Tumor infiltrating lymphocytes	
Low (<50%)	113 (77.4)
High (≥ 50%)	33 (22.6)
Tertiary lymphoid structures around tumor	
Absent	89 (61.0)
Present	57 (39.0)
Tertiary lymphoid structures around tumor	
Low	106 (72.6)
High	40 (27.4)
Germinal center in tertiary lymphoid structures	
Absent	129 (88.4)
Present	(11.6)

* except for age and sizes. SD, standard deviation.

### Association between TILs level of lung adenocarcinomas and various histopathologic factors

The high level of TILs was significantly associated with a high histologic grade (*p* = 0.014), the presence of necrosis in tumor (*p* = 0.042), the presence of TLSs around tumor (*p*<0.001), the abundance of TLSs around tumor (*p*<0.001) and the presence of germinal center in TLSs (*p* = 0.004). However, the presence of a germinal center in TLSs was not correlated with high TILs level among the 57 patients with TLSs (data not shown). Additionally, the high TILs level correlated with absence of lymphovascular invasion, though this was not statistically significant (p = 0.067) (Table 2). In correlation analysis, the TILs level and TLSs abundance showed a positive association (rho = 0.438, p<0.01).

**Table 2:** Clinicopathologic correlation according to level of TILs^+^.

	Low TILs level (<50%) Number (%)*	High TILs level (≥50%) Number (%)*	*P*-value
Age**	64.170±10.350	62.210±9.552	0.333
Size (cm) **	2.872±1.352	2.672±1.207	0.446
Smoking history			0.231
Never-smoker	69 (61.1%)	16 (48.5%)	
Smoker	44 (38.9%)	17(51.5%)	
Histologic grade			**0.014**
Well differentiated	91 (80.5%)	20 (60.6%)	
Moderately differentiated	18 (15.9%)	9 (27.3%)	
Poorly differentiated	4 (3.5%)	4 (12.1%)	
Necrosis			**0.042**
Absent	89 (78.8%)	20 (60.6%)	
Present	24 (21.2%)	13 (39.4%)	
Extracellular mucin production			0.786
Absent	96 (85.0%)	27 (81.8%)	
Present	17 (15.0%)	6 (18.2%)	
Lymphovascular invasion			0.067
Absent	90 (79.6%)	31 (93.9%)	
Present	23 (20.4%)	2 (6.1)	
Lymph node metastasis			0.103
Absent	82 (72.6%)	29 (87.9%)	
Present	31 (27.4%)	4 (12.1%)	
Stage			0.196
Early (stage I and II)	90 (79.6%)	30 (90.9%)	
Advanced (stage III and IV)	23 (20.4%)	3 (9.1%)	
TLSs^++^ around tumor			**<0.001**
Absence	81 (71.7)	8 (24.2)	
Presence	32 (28.3)	25 (75.8)	
TLSs around tumor			**<0.001**
Low	97 (85.8%)	9 (27.3%)	
High	16 (14.2%)	24 (72.7%)	
Germinal center in TLSs			**0.004**
Absent	105 (92.9%)	24 (72.7%)	
Present	8 (7.1%)	9 (27.3%)	
EGFR^+++^ mutation			
No data	6	5	
Absent	48 (44.9%)	14 (50.0%)	0.627
Present	59 (55.1%)	14 (50.0%)	
KRAS mutation			
No data	7	5	
Absent	92 (86.8%)	24 (85.7%)	0.868
Present	14 (13.1%)	4 (14.3%)	

* except for age and sizes.

** t-test was performed and the equality of variances was assumed. SD, standard deviation. The Pearson’s Chi-square test or Fisher’s exact test was used as appropriate for the other variables. EGFR and KRAS mutation was detected by pyrosequencing.

^+^TILs, tumor-infiltrating lymphocytes

^++^TLSs, tertiary lymphoid structures

^+++^EGFR, epidermal growth factor receptor

### Prognostic significance of TILs in lung adenocarcinomas

**Fig 3:**
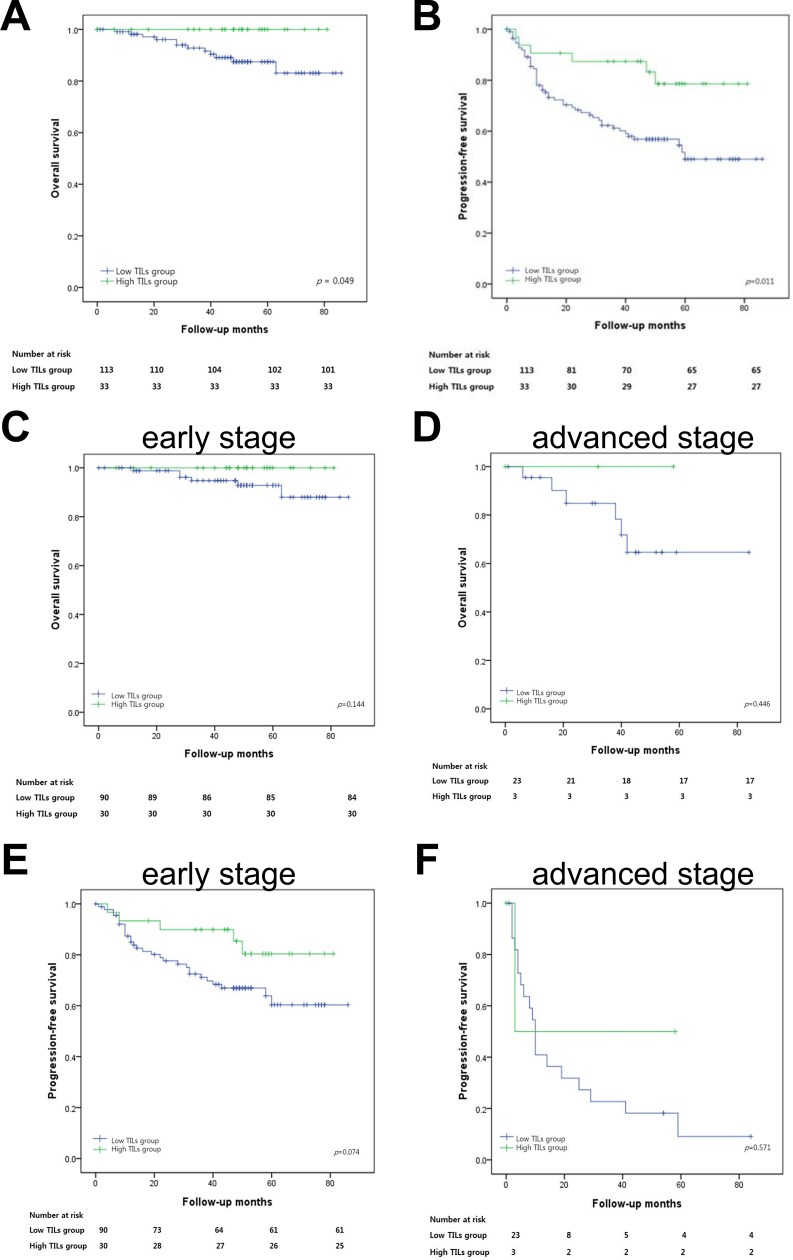
Survival analysis in resected lung adenocarcinoma. (A) Overall survival according to tumor-infiltrating lymphocytes (TILs) level (B) Progression-free survival according to TILs level (C) Overall survival according to TILs level in early stage (stage I and II) disease (D) Overall survival according to TILs level in advanced stage (stage III and IV) disease (E) Progression-free survival according to TILs level in early stage (stage I and II) disease (F) Progression-free survival according to TILs level in advanced stage (stage III and IV) disease.

The median follow-up was 48 months (0–86), and twelve patients died during the follow-up period. Fifty-two patients were found to have either metastasis or recurrence. According to Kaplan-Meier survival analysis, a high level of TILs in lung adenocarcinoma was significantly associated with a better progression-free survival (p = 0.011) and overall survival (p = 0.049). In early stage disease, high TILs level tended to associate with better progression-free survival, though it was not statistically significant (p = 0.078) (Fig 3). On multivariable analysis, high TILs level remained a good prognostic factor for progression-free survival after correcting for factors which were significant on univariate analysis (Hazard ratio: 0.389, 95% confidence interval: 0.161–0.941, *p* = 0.036, Table 3).

**Table 3:** Univariate and multivariable analyses of overall survival and progression free survival.

	Overall survival			Progression free survival					
	Univariate analysis			Univariate analysis			Multivariable analysis*		
	HR**^,^^+^	95% CI^++^	*p*-value	HR	95% CI	*p*-value	HR	95% CI	*p*-value
Age (≥64 yeas vs <64 years)	1.381	0.438–4.355	0.581	1.443	0.843–2.470	0.181			
Histologic grade (moderately & poorly vs well)	0.773	0.168–3.550	0.741	2.286	1.301–4.018	**0.004**	2.034	1.105–3.743	**0.023**
Lymphovascular invasion (present vs absent)	5.300	1.652–17.004	**0.005**	4.901	2.767–8.681	**<0.001**	2.752	1.369–5.531	**0.004**
Stage (III & IV vs I & II)	7.009	2.210–22.228	**0.001**	5.197	2.970–9.094.	**<0.001**	2.627	1.323–5.216	**0.006**
TILs^+++^ (≥50% vs <50%)	0.032	0.000–8.784	0.230	0.353	0.151–0.825	**0.006**	0.389	0.161–0.941	**0.036**

*Multivariable analysis was performed with covariables showing p-value of less than 0.10 in the univariate analyses for multivariable analysis.

**The likelihood ratio test was done to ensure the proportional hazard assumptions in the Cox regression model.

^+^HR, hazard ratio

^++^CI, confidence interval

^+++^TILSs, tumor-infiltrating lymphocytes

## Discussion

There have been various studies to determine the significance of the immune microenvironment in NSCLCs. Most of these have focused on the subpopulation of TILs using immunohistochemistry for various subtypes of immune cells [14–20]. High levels of CD8 positive T-cell infiltration showed positive effects on prognosis in many reports [14, 16, 18, 20], however these studies used different cutoffs to define low and high groups, and some used manual counting while others used digital image analysis. Although TILs assessment with routine H&E sections can be easily applied to the routine workflow of pathology and can facilitate rapid accumulation of knowledge of TILs, there is only limited data addressing generalized TILs based on H&E section evaluation. Kilic et al. reported that a high level of TILs was significantly associated with lower disease-recurrence in large sized NSCLCs [21]. Horne et al. showed that patients with a high intra-tumoral TILs level had better recurrence-free survival in stage IA NSCLCs [22]. Also, intense lymphocytic infiltration was associated with better overall survival and disease-free survival in the discovery and validation set of resected NSCLCs [23]. Feng et al. also showed that a higher density of TILs was correlated with a better postoperative survival time in completely resected stage IIIA NSCLCs using the method previously proposed by Kilic and Horne [24]. However, the methodology for TILs evaluation was briefly described in these studies, although they showed consistent positive impacts. Recently, Rakaee et al. reported that a high level of stromal TILs was associated with better disease-specific survival, overall survival, and disease-free survival [25]. Also, they suggested the methodology of TILs assessment, adapted from the recommendations by an International TILs Working Group of breast cancer, could be applied to NSCLCs.

We evaluated TILs level primarily based on a standardized method from the International Immuno-Oncology Biomarkers Working Group, which also chiefly referred to the International TILs Working Group recommendation. The method from the International Immuno-Oncology Biomarkers Working Group precisely considered the structures and growth patterns of the lung adenocarcinoma including lepidic growth, aerogenous spread, and papillary structures.

A high TILs level was associated with a high histologic grade (*p* = 0.014) in this study. This may be due to a small number of high histologic grades and uneven distribution of histologic grades in our cohort. High level of TILs was an independent good prognostic factor on progression-free survival, however, multivariable analysis for overall survival was not possible, because there were only 12 deaths in our cohort.

We have solely evaluated the prognostic significance of histologic TILs level in lung adenocarcinoma, while others have included NSCLCs including squamous cell carcinoma and the like. Also, this study has additionally avoided the peri-bronchial area in evaluation of TILs to exclude BALTs in this study. These are found in the lungs of fetus and disappear in normal adults [26], though BALTs were found in a small subset of adults in autopsy series [27]. They are also significantly more common in smokers than non-smokers [28].

Considering the prognostic significance of TILs, a better understanding of TILs and the mechanism of TILs recruitment into the tumor is critical in the development of effective immunotherapy. TLS mature dendritic cells are suggested to have a major role in the shaping of the tumor immune contexture in NSCLCs [29, 30]. The density of mature dendritic cells, which are specific markers of TLSs, was positively correlated with the density of TILs, especially CD4 positive and T-bet positive Th1 cells in early stage NSCLCs [29]. Also, the density of dendritic cells was significantly associated with strong infiltration of T cells and high density of mature dendritic cells associated with long-term survival in completely resected NSCLCs [18]. Taken together, these data suggest the role of TLSs in T-cell recruitment in NSCLCs. This is in accordance with our data which shows that TILs level and TLSs abundance have positive association (rho = 0.438, p<0.01).

Although two pathologists reviewed the slides, we used the multi-head microscope to reach a consensus. We could not assess the inter-observer variability or reproducibility in this study. Also, comparison with morphometric analysis of histologic TILs level should be performed in future studies.

## Conclusion

Histologic assessment of TILs with H&E section in lung adenocarcinoma has prognostic value. The level of TILs was significantly associated with abundance of TLSs around the invasive tumor. Also, the standardized method proposed by the International Immuno-Oncology Biomarkers Working Group could be useful in routine surgical pathologic practice.
